# Development and Characterization of 50 nanometer diameter Genetically Encoded Multimeric Nanoparticles

**DOI:** 10.1101/2024.07.05.602291

**Published:** 2024-07-07

**Authors:** Cindy M. Hernandez, David C. Duran-Chaparro, Trevor van Eeuwen, Michael P. Rout, Liam J. Holt

**Affiliations:** 1. Institute for Systems Genetics, New York University School of Medicine, New York, 435 E 30th Street NY 10016, United States; 2. Laboratory of Cellular and Structural Biology, The Rockefeller University, New York, NY10065,

## Abstract

The mechanisms that regulate the physical properties of the cell interior remain poorly understood, especially at the mesoscale (10nm-100nm). Changes in these properties have been suggested to be crucial for both normal physiology and disease. Many crucial macromolecules and molecular assemblies such as ribosomes, RNA polymerase, and biomolecular condensates span the mesoscale size range. Therefore, we need better tools to study the cellular environment at this scale. A recent approach has been to use genetically encoded multimeric nanoparticles (GEMs), which consist of self-assembling scaffold proteins fused to fluorescent tags. After translation of the fusion protein, the monomers self-assemble into bright and stable nanoparticles of defined geometry that can be visualized by fluorescence microscopy. Physical properties of the cell can then be inferred through analysis of the motion of these particles, an approach called nanorheology. Previously, 40nm-GEMs elucidated TORC1 kinase as a regulator of cytoplasmic crowding. However, extremely sensitive microscopes were required. Here, we describe the development and characterization of a 50 nm diameter GEM that is brighter and probes a larger length scale. 50nm-GEMs will make high-throughput nanorheology accessible to a broader range of researchers and reveal new insights into the biophysical properties of cells.

## Introduction

Eukaryotic cells are crowded with macromolecules, membrane-bound organelles, and a dynamic cytoskeleton^1,2^. The diffusion of particles within this complex environment is decreased by macromolecular crowding but increased by metabolic activity, polymerization and depolymerization (especially of cytoskeletal networks), and the activities of molecular motors, such as myosins, kinesins, and helicases.

It is of particular importance to understand the physical properties of the intracellular environment at the mesoscale (~10 to 1000 nanometers diameter) - because a large fraction of the space in the cell, is taken up by particles of this size (e.g. nucleosomes are ~ 10 nm, ribosomes are ~30 nm, mRNA ribonucleoproteins complexes are ~100 nm). Most monomeric proteins are in the nanometer (1–5 nm) size range^3,43–5^. However, many proteins also assemble into mesoscale complexes^6,7^. Furthermore, recent experiments suggest that at least 18% of proteins in the *Xenopus laevis* cytoplasm are organized into mesoscale biomolecular condensates of predominantly 100 nanometer diameter^8^. Therefore, we need better technologies and approaches to understand how the intracellular environment influences mesoscale particles.

Some of the most successful characterizations of the cell interior to date have employed **nanorheology**, which is the inference of biophysical properties from observations of the motion of tracer particles^9^. A major challenge is the shortage of methods to introduce probes for nanorheology into cells. Approaches such as microinjection of tracer particles into cells can damage cells and dilute the cytoplasm. Furthermore, these approaches are labor intensive and low-throughput.

To address these challenges, our lab has previously reported the development of Genetically Encoded Multimeric (GEM) nanoparticles of 20 and 40 nanometer diameter (20- and 40nm-GEMs respectively)^10^. We based these probes on scaffold proteins that homomultimerize: single gene products self-assemble into icosahedral structures of precise geometry. We fused these scaffold proteins to fluorescent proteins yielding bright, stable nanoparticles. We can use different scaffolds to make GEMs of different sizes, allowing us to probe the physical properties of the cell at different length-scales. GEMs are constantly expressed, solving all of the problems mentioned above: no dilution occurs; the cortex and membrane are not disrupted; and every cell contains nanoparticles, enabling acquisition of data from thousands of cells. Moreover, GEMs are well tolerated^11,12^, allowing long-term studies and high content genetic screens. GEMs have defined geometry and size allowing precise physical interpretation of their motion. They are bioorthogonal, and therefore unlikely to be subject to regulated interactions with the cell. GEMs are extremely easy to use: no microinjection or laborious sample preparation is required, enabling the rheological characterization of many species for the first time. For example, it is impossible to micro-inject microorganisms that have cell walls, including plants or yeasts, but GEMs can be easily introduced into these organisms^12–15^. However, expansion of the available probes within the mesoscale range would provide deeper insights into the biophysical properties of the cell interior. Furthermore, previous GEMs were challenging to use at sufficient temporal resolution (50–100 Hz) because highly sensitive microscopes are required to achieve sufficient signal at these rapid frame-rates. Thus, we have expanded our nanorheology probe toolkit by developing a 50 nm diameter GEM derived from *Quasibacillus thermotolerans*^16^. These new 50nm-GEMs are brighter, easier to use, and probe the biophysical properties of the cell at the 50 nm length-scale.

## Results

### 50nm-GEMs are brighter than 40nm-GEMs

**Figure 1.**
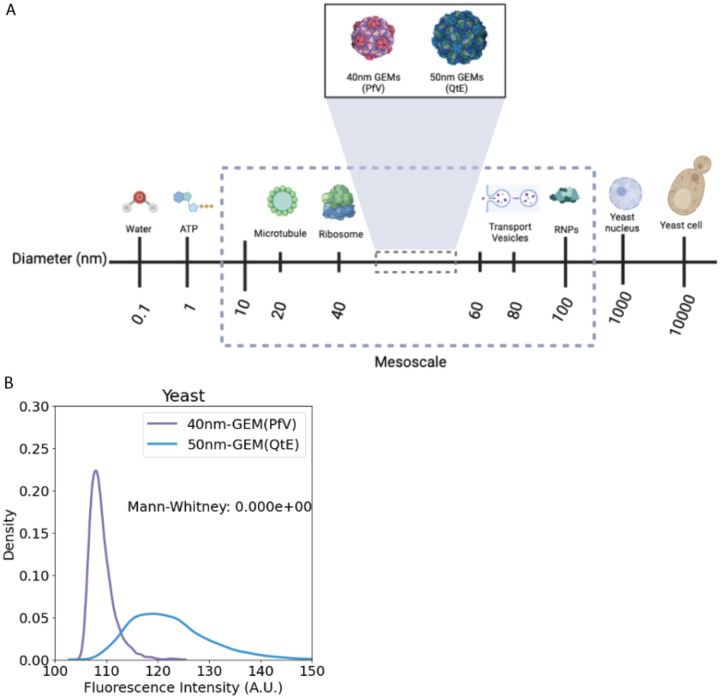
(A) Sizes of various biomolecules in relation to GEMs. (B) Average brightness of single particle of 40nm-GEMs and 50nm-GEMs expressed in *S. cerevisiae*. The median brightness for 50nm-GEMs is significantly higher than that of 40nm-GEMs.

In this study, we used the encapsulin domain from *Quasibacillus thermotolerans*, as the scaffold for GEM assembly, and fused fluorescent tags at the C-terminus of this domain. Previous structural analysis showed that the encapsulin self-assembles into a 240-subunit icosahedral compartment with a diameter of 42 nm^16^. We predicted that this protein would assemble with approximately 50 nm diameter due to the addition of a cloud of GFP at the surface of the encapsulin. This 50nm-GEM would probe a crucial length-scale relevant to many bioregulatory mechanisms (Fig. 1A). In addition, 50nm-GEMs should contain 240 fluorophores and therefore be brighter than previous GEMs. Indeed, Figure 1B shows that 50nm-GEMs are substantially brighter.

### CryoEM Structural Analysis of 50nm-GEMs

To understand the size and nature of the 50 nm-GEM particles, we turned to native purification and structural characterization by cryo-Electron Microscopy single particle analysis (Cryo-EM SPA). To enable purification of 50nm-GEMs, we fused a FLAG-tag sequence to the C-terminus of the fluorescent tag^17^. 50nm-GEMs were expressed from the HIS3 promoter in *Saccharomyces cerevisiae*, cells were lysed by cryogenic milling^18^, GEMs were purified by affinity capture of the FLAG-peptide, purified 50nm-GEMs were imaged by Cryo-EM SPA.

**Figure 2.**
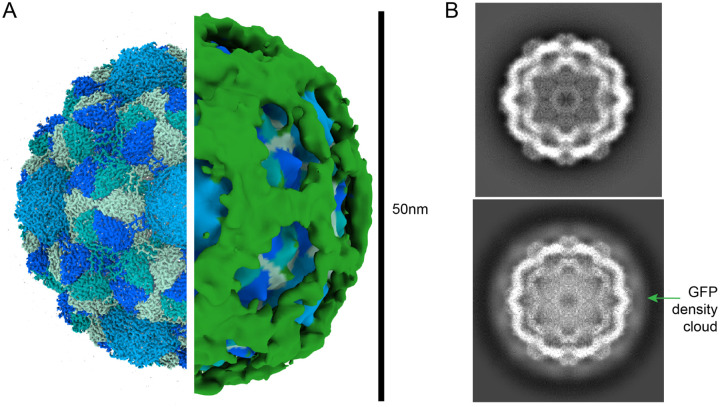
Cryo-EM single particle analysis of 50 nm-GEMs. **A)** High resolution map of the GEM encapsulin cage (left) and low-pass filtered GEM particle with GFP density (green, right) reveal a GEM with a diameter of 50 nm (scale bar right). **B**) 2D projection of *Quasibacillus thermotolerans* two-component encapsulin-based iron storage compartment (EMDB:9383, top) and the 50 nm-GEM (this study, bottom) shows a clear density outside of the encapsulin cage ascribable to GFP.

SPA of endogenously purified GEMs yielded a 2.85-angstrom reconstruction, grossly similar to previously characterized recombinant *Quasibacillus thermotolerans* two-component encapsulin-based iron storage compartment^16^. Like the previously studied, recombinant encapsulin, the endogenously purified GEMs form a ~42 nm internal scaffold with icosahedral symmetry and T = 4 topology (Figure 2A). The four-protomer asymmetric unit from the endogenous GEM and the recombinant *Quasibacillus thermotolerans encapsulin* are remarkably similar with an all atom root mean square deviation (RMSD) of 0.652 angstroms.

The high-resolution GEM reconstruction, while providing a detailed structure of the scaffold, lacked significant density for the attached GFP proteins, likely due to their flexibility. To obtain information about the full hydrodynamic radius of the 50 nm-GEM, we repeated the refinement with symmetry relaxation. This approach yielded a 4-angstrom reconstruction with an obvious density that could be ascribed to GFP (Figure 2A). The GFP tags form an apparent cloud around the encapsulin core scaffold, leading to a particle with an approximate diameter of 51 nm (Figure 2A). Projections of the previously studied *Quasibacillus thermotolerans* encapsulin do not show this GFP cloud (Figure 2B). Additionally, cross-sectional analysis of the two maps shows the 50 nm-GEM particle lacks density previously ascribed to the cargo protein of the *Quasibacillus thermotolerans* iron storage compartment. This map demonstrates that endogenously expressed GEMs are primarily assembled as particles with a particle radius consistent with our expectations of 50 nm.

### Nanorheological analysis of the *Saccharomyces cerevisiae* cytoplasm using 40nm-GEMs and 50nm-GEMs

**Figure 3.**
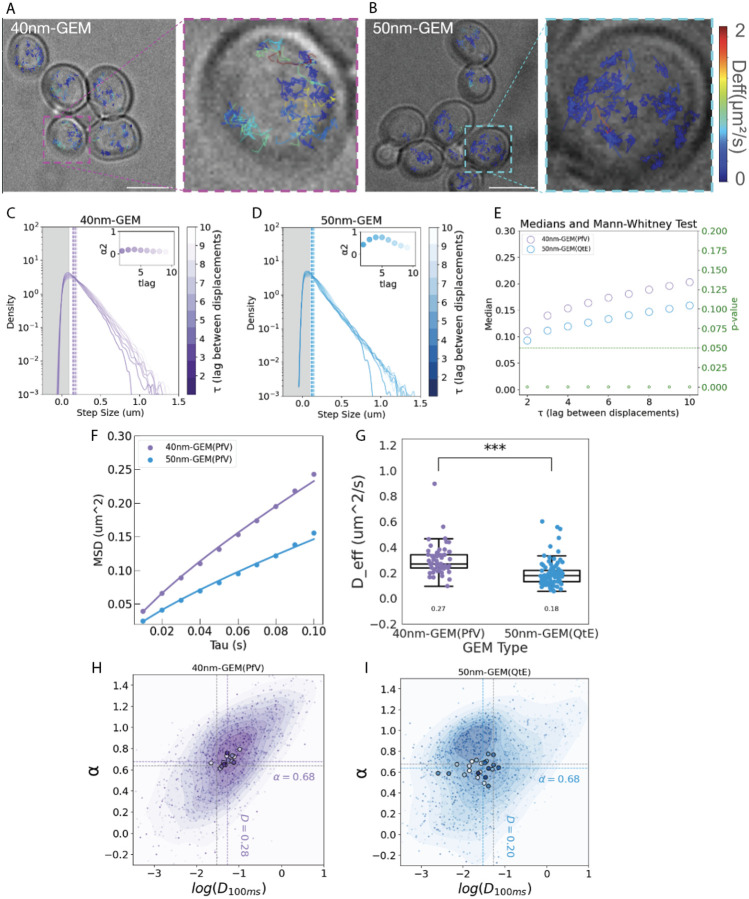
50nm-GEMs show lower effective diffusion compared to 40nm-GEMs in *S. cerevisiae*. **(A,B)** Rainbow tracks of 40nm-GEMs (PfV) and 50nm-GEMs (QtE) overlaid with brightfield images of yeast. We used a jet color scale to indicate the Effective Diffusion Coefficient. Blue represents the lowest diffusion coefficient and red the highest diffusion coefficient, demonstrating that 50nm-GEMs move slower than 40nm-GEMs. **(C,D)** Step Size Distributions for the two types of GEMs. The y axis is in log scale. Lower t_lags are plotted darker. Gray area on the left represents the pixel size as a measure of uncertainty. The inset shows the *α*2 parameter for the different t_lags. The sudden drop on t_lag 1 is probably an artifact due to the linking of the trajectories when tracking. Distributions appear to be Gaussian. **(E)** Medians of the step size distribution for different t_lags. 50nm-GEMs have lower medians than 40nm-GEMs. The p-value comes from the Mann-Whitney test on the median and is reported on the right y axis. All the differences in the median are statistically significant. **(F)** Mean squared displacement vs tau plot for the range between 10 and 100 ms. 40nm-GEMs show a higher slope. **(G)** Effective Diffusion Coefficient. Each dot represents individual cells. The stars represent the p-value for the Mann-Whitney test on the medians. Values for median diffusivity are displayed under each boxplot. 50nm-GEMs show lower Diffusion Coefficients than 40nm-GEMs. The *α*-logD plot **(H,I)** shows the anomalous exponent (*α*) and the logarithm of the diffusion coefficient (logD). The blue dots represent individual tracks, and black dots represent the median track values per cell. The color map represents the Kernel Density Estimation (KDE) which accounts for the spatial density of the data points. Darker shades in the KDE represent a higher concentration of data points around that value. For 50nm-GEMs, a high trajectory spread is observed due to cell heterogeneity. In contrast, 40nm-GEMs exhibit less heterogeneity and a narrower data spread. Moreover, the dashed line at *α* = 1 indicates the trajectory reference point for Brownian motion. 40nm-GEMs trajectories are centered around *α*=1, indicating Brownian motion of these particles. In contrast, 50nm-GEM trajectories appear as two subpopulations, with one population centering around *α*=1, and the other centering around lower *α* values, indicating subdiffusive motion.

Both 40nm-GEMs and 50nm-GEMs formed bright and stable fluorescent nanoparticles when expressed in *Saccharomyces cerevisiae* (Fig. 3A, B). To enable nanorheology analysis, we imaged these particles at high temporal resolution (100 Hz) in log-phase yeast cells. Four second GEM movies were taken at a frequency of 100Hz (period = 10ms, 400 frames.) We compared single-particle trajectories between 50nm-GEMs and 40nm-GEMs (Fig. 3). The particle trajectories were captured using Mosaic in FIJI^19^ and analyzed using the GEMspa software package^20^. Projections of trajectories over time indicated that typical 50nm-GEM trajectories explored less space than 40nm-GEMs (Fig. 3A, B).

The step size distribution (Fig. 3C, D) is the probability distribution of all of the trajectory displacements. Each trajectory has a set of displacements calculated as the difference between consecutive positions. The ensemble of all trajectory displacements is then binned, and the step size distribution calculated. The step size distribution reveals lower medians for 50nm-GEMs compared to 40nm-GEMs at all time delays (Fig. 3E), as expected for larger particles.

Normal, Brownian diffusion should result in a Gaussian step size distribution. Deviation from this Gaussian behavior is characterized by the *α*2 parameter. This parameter is close to zero across all time lags (insets in Fig. 3C, D), indicating relatively Gaussian behavior. Notably, there is no indication of barriers impeding GEM motion, which would manifest as sharp decreases in all step size distributions

Figure 3F shows the mean and ensemble averaged mean squared displacement (MSD) of particles as a function of time delay (tau). Pure Brownian motion is indicated by a linear relationship between MSD and tau, while subdiffusive motion is characterized by an *α* value less than one. Subdiffusion can arise for multiple reasons including interaction with the environment, confined motion, or spatial heterogeneity. The systematic downward deviation of this plot indicates overall subdiffusive behavior.

Next, By fitting the MSD versus tau data to Einstein’s Diffusion Equation^21^, we derived an effective diffusivity (D), which is a simple quantitative measure of the GEM nanoparticle dynamics in-vivo. Figure 3G shows the median diffusivities of 40nm-GEMs (left) and 50nm-GEMs in single *S. cerevisiae* cells. There is significant variability in the physical properties of single *S. cerevisiae* cells, similar to previous observations in the fission yeast *Schizzosacharomyces pombe*^22^, but the median diffusivity across all cells is significantly lower for 50nm-GEMs.

We next determined both diffusivities and the anomalous exponent of all trajectories by linearizing the anomalous diffusion equation using a logarithmic transformation followed by fitting a linear regression to the transformed data. Figures 3H and I show plots of log_10_ D versus *α* for 40nm-GEMs and 50nm-GEMs respectively. This analysis allows us to explore both parameters of the anomalous diffusion equation without assuming a specific type of motion.

Each trajectory’s anomalous diffusion parameters were fitted and then plotted in a two-dimensional space as small dots, while the median values per cell are represented by larger dots. We used Kernel Density Estimation (KDE) to group these data points. For Brownian diffusion, these distributions should center around *α* = 1. The major distribution for both particles follows this expectation, but an additional cluster of trajectories with lower *α* is also apparent for 50nm-GEMs (Fig 3I). Thus, 50nm-GEMs seem to reveal that the cytoplasmic environment is more heterogeneous at the 50 nm length-scale than the 40 nm length scale.

## Conclusion

Here we describe the development and characterization of a 50 nanometer diameter GEM nanoparticle (50nm-GEMs) that will be a useful additional tool for biophysical analysis of the yeast cytoplasm. We have also developed versions of this nanoparticle for use in mammalian cells that will be reported shortly in an update to this initial bioRxiv.

## Methods

### Yeast strains

All *S. cerevisiae* strains constructed for and used in this study are listed in Table 1. Unless otherwise stated, strains were grown at 30 °C in synthetic complete media + 2% dextrose (SCD) according to standard Cold Spring Harbor Protocols. Yeast strains were constructed using standard molecular genetic methods, and verified by fluorescence microscopy for GEM expression. In preparation for imaging via fluorescence microscopy, cells were grown at 30°C in a rotating incubator for 4–5 hours to reach log phase at O.D 600, at which point they were diluted for overnight growth to reach an O.D 600 0.2 and 0.4.

### Construction of 50nm-GEM and 40nm-GEM plasmids.

The open reading frame (ORF) encoding the *Quasibacillus thermotolerans* (QtE) encapuslin protein based on the published crystal structure (https://www.rcsb.org/structure/6NJ8) was codon optimized for yeast expression and synthesized as an IDT gene block (http://www.idtdna.com/pages). The 50nm-GEM yeast expression vector was constructed by Gibson assembly into a pRS305 vector, with fusion of the 5’ end of the QtE-encapsulin ORF to a homologous sequence of the yeast INO4 promoter, and at the 3’ end, via a 6xGly-Ser linker, with the the ORF of the T-Sapphire fluorophore (pLH1976: pRS305- PINO4-QtE-GS-Sapphire). The mammalian expression vector was assembled by subcloning the QtE-GS-T-Sapphire gene cassette into a pLVX lentiviral backbone, with transcription directed by the mammalian pUBC promoter.

For insertion of the FLAG tag, the FLAG and GFP sequence was cloned together with the GFP sequence into a yeast expression vector containing the scaffold sequence for 50nm-GEMs (QtE) by Gibson Assembly. The plasmid was stored in bacteria glycerol stocks and plasmids generated in this study are deposited at AddGene under the accession number XXXX>.[still editing this section]

**Table:** 

Plasmid ID	Addgene ID		Origin
pLH497		pRS305-pINO4-PfV-GS-Sapphire	Liam Holt Lab
pLH1976		pRS305-pINO4-QtE-GS-Sapphire	Liam Holt Lab
pLH2608		pRS305-pINO4-PfV-GS-Sapphire-GS-FLAG	Liam Holt Lab

### Affinity-purification of 50 nm-GEMS from S. cerevisiae

We adapted a previously published method for the isolation of endogenous whole NPCs from *S. cerevisiae* (PMID:35412228) to purification of natively expressed GEMs. Briefly, strains harboring FLAG-tagged 50nm-GEMs and 40nm-GEMs incorporated at the *LEU2* locus were grown in YPD media at 30°C until mid-log phase (~3×10^7^ cells/ml), harvested, frozen in liquid nitrogen and cryogenically lysed in a planetary ball mill PM 100 (Retsch) (http://lab.rockefeller.edu/rout/protocols). Affinity purification was performed using anti-FLAG or anti-GFP dynabeads (Thermo) in clarified lysate of cryo-milled yeast in resuspension buffer (20 mM HEPES/KOH pH 7.4, 500 mM sodium chloride, 0.1% (w/v) Triton X-100, 1 mM DTT, 10% (v/v) glycerol, 1/500 (v/v) Protease Inhibitor Cocktail (Sigma)). GEMs were either eluted by denaturing for SDS-PAGE analysis or FLAG peptide for structure characterization. For SDS-PAGE analysis, affinity captured GEMs were eluted from beads by addition of 20 μl of 1x LDS (lithium dodecyl sulfate) loading buffer (Thermo Fisher) and vortexing for 10 minutes at room temperature. Eluted GEMs were run on an NuPAGE 4–12% gel for 60 minutes and analyzed by Coomaisse.

For structural studies, GEMs were eluted by nutating beads with elution buffer (20 mM HEPES/KOH pH 7.4, 500 mM sodium chloride, 0.1% (w/v) Tween-20,1 mM DTT) supplemented with 1mg/mL of 3xFLAG peptide for 20 minutes at room temperature. Eluted GEMs were further analyzed by gradient sedimentation through a 10–60% glycerol gradient with a SW60 Ti (Beckman) rotor. Gradients were spun at 32,000 RPM for 2 hours and 45 minutes and fractionated and profiled using BioComp piston fractionator and Triax profiler. Peak fractions, as determined by fluorescence signal, were pooled and concentrated.

### Cryo-EM analysis of 50 nm-GEMs

Grids for single particle analysis were made by incubating R2/2 300 mesh Quantifoil grids with thin carbon support on 20 μL drops from concentrated 50 nm-GEMs for 10 minutes in a humidity chamber to allow for particle adhesion. Grids were then washed with wash buffer (20 mM HEPES/KOH pH 7.4, 150 mM sodium chloride, 1 mM DTT) ensuring to keep the back of the grid dry. A final volume of 3 μL of wash buffer was applied to grids in the Vitrobot (ThermoFisher) humidity chamber. Grids were blotted for 3 seconds at blot force 3 and plunged using a Vitrobot.

For SPA analysis, 50 nm-GEM grids were imaged on a Titan Krios (ThermoFisher) with spherical aberration (Cs) correction and BioQuantum energy filter (Gatan), using a K3 direct electron detector operated in super-resolution mode. Movies of 40 frames were collected at a nominal pixel size of 0.54 angstroms with a target electron flux of 51 e^−^/Å^2^. Movies were imported into cryoSPARC v4.4.1 (PMID: 28165473) and binned by 2 during patch motion correction for a final pixel size of 1.08 angstroms. Motion corrected micrographs were CTF corrected with CtfFind 4.1.14 (PMID: 26278980) and were manually filtered for quality of CTF fit, total micrograph motion and ice quality, yielding a dataset of 7,323 micrographs.

Due to their significant size, 50 nm-GEMs were easily distinguished and picked by blob picking in cryoSPARC, yielding 157,358 particles that were subject to 2D classification. Even in initial 2D classes, fuzzy density outside of the clearly detailed core scaffold was observed, suggesting C-terminally linked GFPs were oriented on the external surface and provided extra volume to the 50 nm-GEM. Only 2D classes containing clearly intact GEMs, representing 28,772 particles, were selected and analyzed in 3D. An *ab initio* generated model from the data by stochastic gradient descent (SGD) was remarkably similar to the previously published (EMDB:9383) *Quasibacillus thermotolerans* iron storage compartment with clear icosahedral symmetry, so this was used for downstream processing. Iterations of homogeneous refinement, local and global CTF refinement and local motion correction yielded a final reconstruction of 2.85 angstroms. The map was post-processed by manually determined B-factor sharpening (B=−100) and local resolution was determined using MonoRes (PMID: 29395788). To retrieve density for the C-terminal GFPs since they represent a breakage of the icosahedral symmetry due to their flexible nature (PMID: 29439140), homogeneous refinement with pose marginalization and limited alignment was performed. This yielded a reconstruction with a nominal resolution of 4.6 angstroms with clearer density we ascribe to C-terminal GFP (Figure 2A). Measurement of this reconstruction radius yielded an average of 51 nm.

To model the core scaffold of the 50 nm-GEM, the atomic model from the encapsulin iron storage compartment from *Quasibacillus thermotolerans* (PDB:6NJ8) was fit as a rigid body into the 2.85 angstrom map in UCSF ChimeraX (PMID:37774136). This was then used as the basis for interactive molecular dynamics flexible fitting (IMDFF) with ISOLDE (PMID: 29872003). The model was finally refined in phenix using real space refinement (PMID: 29872004), yielding a model with a map-to-model correlation of 3.1 angstroms at FSC=0.5. Due to the poor density, we opted not to build an atomic model for the GFP. Structural figures were generated using a combination of UCSF ChimeraX (PMID:37774136) and eman2 (PMID: 16859925).

### Microscopy of yeast cells

For imaging yeast, cells were cultured to log phase at OD600 0.4 in synthetic complete media (2% Dextrose). 384 glass-bottom plates were coated with Concanavalin-A (ConA). Using a Nikon Ti Eclipse Microscope equipped with a Hamamatsu camera, frames were captured every 10 ms over a duration of 400 frames. Fluorescence was observed using lasers with specifications detailed in the supplementary material.

### Further details on analysis of trajectories

We determined both diffusivities and the anomalous exponent of all trajectories by linearizing the anomalous diffusion equation using a logarithmic transformation followed by fitting a linear regression to the transformed data. Figures 3H and I show plots of log_10_ D versus *α* for 40nm-GEMs and 50nm-GEMs respectively.

The next phase involves using Kernel Density Estimation (KDE) to group these data points. The technical details of equation solving and KDE implementation are further explored in the methodology section of the analysis. Rather than using centroids, the resulting medians of the distributions are plotted. This approach is particularly useful during the single-cell analysis, as it provides a clearer depiction of changes in diffusivity.

The diffusion form is estimated from these plots. For Brownian diffusion, a distribution centered around \(\*α* = 1 \) is expected, with the KDE plot revealing the spread around this central value. This analysis sets the stage for characterizing various motion types, such as macromolecular crowding, which deviates from Brownian motion and displays distinctive patterns. For example, confined motion is often associated with a lower diffusion coefficient.

We analyze the motion of each trajectory by fitting the MSD vs. tau plot to a line. The parameters of this line correspond to the *α* value (slope) and diffusion coefficient (intercept).

The one dimensional Einstein equation is:

$$\langle \delta x^{2}\rangle =2D_{eff}t$$

Which for two dimensions is:

$$\langle \delta r{\left(x,y\right)}^{2}\rangle =4D_{eff}t$$

### Software and Algorithms

The following software and algorithms were employed in the analysis: - ImageJ - Fiji - Mosaic - Cellpose - GEMspa (8) The code used in this study is available at https://github.com/liamholtlab

## Figures and Tables

**Table 1.** Yeast Strain Information

Strain ID	Genetic Background	Modification	Origin
LH4501	BY4741	pINO4:: pINO4-Pfv-Sapphire-LEU2	Liam Holt Lab
LH4580	BY4741	pINO4:: pINO4-QtE-Sapphire-LEU2	Liam Holt Lab
LH4832	BY4741	pHis3::pHis3-QtE-GFP-FLAG-URA3	Liam Holt Lab
LH4348	BY4741	pINO4::pINO4-PfV-GFP-FLAG-LEU2	Liam Holt Lab

## Data Availability

For additional information and requests, please contact Liam Holt (Liam.Holt@nyulangone.org) or Cindy Hernandez (Cindy.Hernandez@nyulangone.org).
